# The long-range transport of Pinaceae pollen: an example in Kraków (southern Poland)

**DOI:** 10.1007/s10453-016-9454-2

**Published:** 2016-09-23

**Authors:** Kazimierz Szczepanek, Dorota Myszkowska, Elżbieta Worobiec, Katarzyna Piotrowicz, Monika Ziemianin, Zuzanna Bielec-Bąkowska

**Affiliations:** 1 Institute of Botany, Jagiellonian University, Kraków, Poland; 2 Department of Clinical and Environmental Allergology, Jagiellonian University Medical College, Kraków, Poland; 3 W. Szafer Institute of Botany, Polish Academy of Sciences, Kraków, Poland; 4 Institute of Geography and Spatial Management, Jagiellonian University, Kraków, Poland; 5 Faculty of Earth Science, University of Silesia, Sosnowiec, Poland

**Keywords:** *Abies*, *Picea*, *Pinus* pollen, Meteorological conditions, Back-trajectory analysis

## Abstract

High Pinaceae pollen concentrations in the air and on the surface of puddles before the main pollen season started were observed in Kraków (southern Poland) in May 2013. The paper presents the results of detailed studies of the composition and source of the “yellow rain” in 2013, and as a comparison, the Pinaceae pollen concentrations and samples collected from the ground surface in 2014 were considered. The air samples were collected using the volumetric method (Hirst-type device), while pollen grains sampled from the ground surface were processed using a modified Erdtman acetolysis method. Finally, all samples were studied using a light microscope. In 2013, the period of higher *Abies*, *Picea* and *Pinus* pollen concentrations was observed from the 5 to 12 of May, earlier than the main pollen season occurred. The presence of rainfall on the 12 and 13 of May 2013 caused the pollen deposition on the ground surface, where the prevalence of Pinaceae pollen was found. The synoptic situation and the analysis of the back-trajectories and air mass advection at the beginning of May 2013 indicated that Pinaceae pollen grains could have been transported from Ukraine, Romania, Hungary and Slovakia. In contrast, Pinaceae pollen grains deposited on the ground surface as a “yellow” film in May 2014, originated from local sources.

## Introduction

There is an increasing interest in the long-range transport of small-sized biological material, because of its negative influence on the environment due to the expansion of the biogeographical ranges of different organisms and on human health due to the dispersion of allergenic pollen and the transport of pathogens (Belmonte et al. 2008). Many studies have been already published concerning the long-range transport of airborne pollen grains: (1) tree pollen transport (Środoń 1960; Peeters and Zoller 1988; Hjelmroos 1991; Welch et al. 1991; Cabezudo et al. 1997; Hicks and Isaksson 2006; Skjøth et al. 2007; Belmonte et al. 2008; Rousseau et al. 2008), (2) grass pollen flow (Smith et al. 2005) and (3) weed pollen transport (Makra and Pálfi 2007; Smith et al. 2008; Kasprzyk et al. 2011; Šikoparija et al. 2013).

There are still very few reports on the Pinaceae pollen transport through the air, although both pollen and seeds of conifers can be transferred with air masses far away from the source of their production and release (Benkman 1995). Hesselman (1919) indicated that pine pollen was transported by air masses from the forests in Scandinavia up to 30–35 km distance. Harmata and Olech (1991) found Pinaceae pollen in the air samples collected along the transect from the South Shetland Islands to Gdynia on the Baltic Seacoast of Poland. Furthermore, Dyakowska (1948) reported that pine and spruce pollen was found 600–1000 km far away from Pinaceae forests, while at a later stage she suggested a limitation range for pine pollen shed to be 74.7 km (Dyakowska 1959).

Pollen of the pine family is produced in copious amounts, but is not generally considered to be allergenic (D’Amato et al. 2007). However, people exposed to coniferous pollen in large quantities can show a irritation reaction, with clinical symptoms similar to typical allergic manifestations (Weber 2014). Knowledge of Pinaceae pollen seasons is useful for warning people of the potential airborne irritant and for explaining the phenomenon of “yellow rain”, which is related to the pollen transfer within the atmosphere from the distant source of pollen release.

**Fig. 1:**
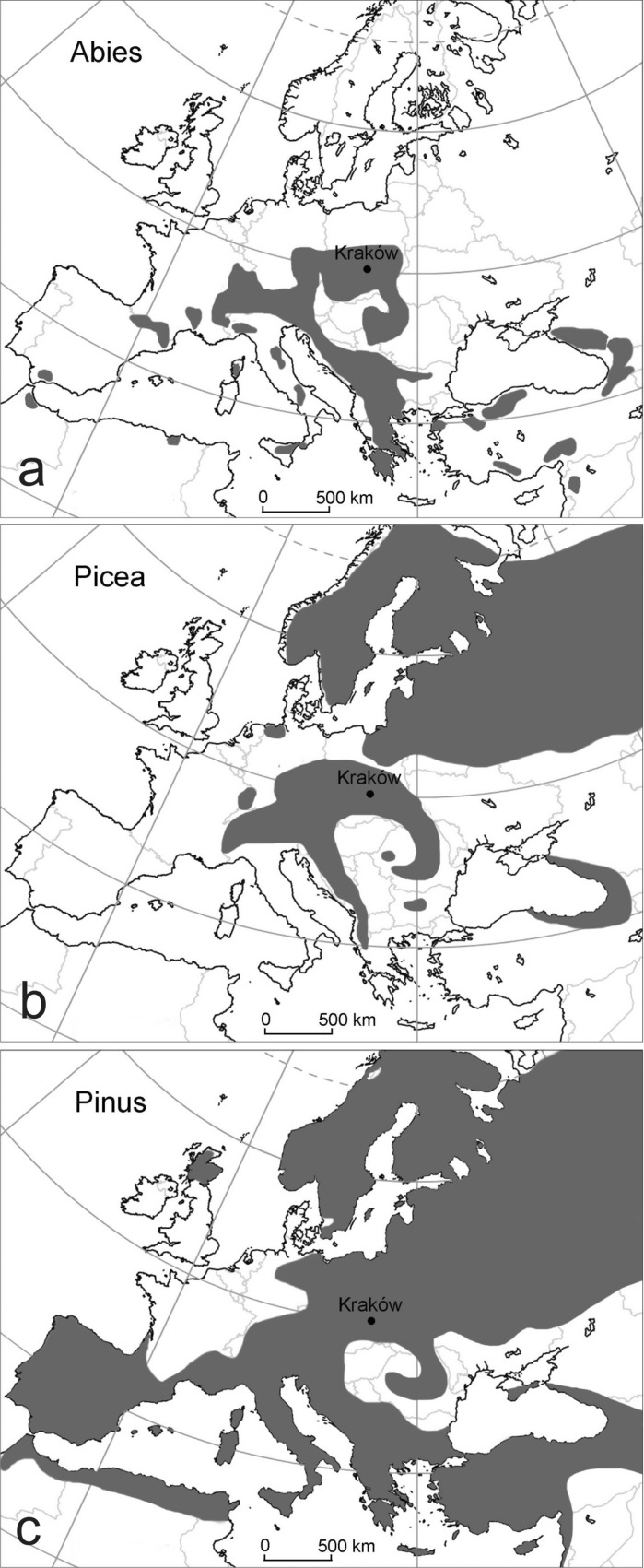
Simplified map of distribution of the genera: *Abies* (**a**), *Picea* (**b**) and *Pinus* (**c**) in Europe (according to Meusel et al. 1965; Krüssmann 1972)

Pinaceae family includes 9 genera and over 210 species of trees (rarely shrubs) native to northern temperate regions (Seneta and Dolatowski 2000), and the detailed information on conifers distribution in Europe is presented in Table 1 and Fig. 1. In Poland, *Pinus sylvestris* L., the most common species of the Pinaceae family occurs in the entire country, while *Abies alba* Mill. occurs in the southern part of the country and *Picea abies* (L.) H. Karst. almost in the whole country, except at the north-western region (Zając and Zając 2001). The intensive “pollen rain” of conifers in the middle of May in Poland is visible in the water bodies, slopes, ground and cars as a yellow film (Weryszko-Chmielewska 2007). Among the Pinaceae family, especially *Pinus* and *Picea* genera produce large quantities of pollen equipped with airbags and that is why they are easily carried by the wind (Faegri and Iversen 1989). Pollen is also detected in the air samples downloaded by the volumetric samplers. Long-term aeropalynological studies performed in Poland indicated that *Pinus* and *Picea* seasons classified by Szczepanek (1994) as the “short” seasons coincided in time within the years of observations.

**Table 1:** Species of the genera *Abies*, *Picea* and *Pinus* occurring in Europe and their ranges (according to Meusel et al. 1965; Krüssmann 1972)

Species	Native range
*Abies alba* Mill.	The most common species of fir; mountains of Europe, from the Pyrenees north to Normandy, east to the Alps and the Carpathians, Slovenia, Croatia, Bosnia and Herzegovina and south to southern Italy and northern Serbia
*Abies borisii*-*regis* Mattf.	Mountains of the Balkan Peninsula in Bulgaria, northern Greece, the Republic of Macedonia, Albania and Serbia
*Abies cephalonica* Loudon	Mountains of Greece
*Abies nebrodensis* (Lojac.) Mattei	Northern Sicily
*Abies nordmanniana* (Steven) Spach	Mountains west and east of the Black Sea, in Turkey, Georgia, Russian Caucasus and northern parts of Armenia
*Abies pinsapo* Boiss.	Southern Spain and northern Morocco
*Picea abies* (L.) H. Karst.	The most common species of spruce; Central and Eastern Europe
*Picea obovata* Ledeb.	Northern Europe
*Picea omorika* (Pančić) Purk.	Endemic to the eastern Bosnia and Herzegovina
*Picea orientalis* (L.) Link	Caucasus and adjacent north-east Turkey
*Pinus cembra* L.	Alps and Carpathian Mountains of central Europe
*Pinus halepensis* Miller, *Pinus nigra* J. F. Arnold, *Pinus pinaster* Aiton, and *Pinus pinea* L.	Mediterranean region
*Pinus heldreichii* H. Christ	Mountainous areas of the Balkans and southern Italy
*Pinus mugo* Turra	High elevation habitats from south-western to central Europe
*Pinus peuce* Griseb.	Mountains of Macedonia, Bulgaria, Albania, Montenegro, Kosovo, the extreme south-west of Serbia, and the extreme north of Greece
*Pinus sylvestris* L.	The most common species of pine, ranging from western Europe to eastern Siberia, south to the Caucasus Mountains and Anatolia, and north to well inside the Arctic Circle in Scandinavia

In 2013, the high *Abies* pollen concentrations in the air and on the surface of puddles in Kraków were observed, although *Abies* pollen has never been noted in such abundance in Kraków since last decade (2003–2012). High pollen concentrations of *Abies* and other Pinaceae genera (*Pinus*, *Picea*) were detected before the pollination period started, which was also confirmed by phenological observations taken in the green urban areas and nearby forests. Thus, no local coniferous trees could be responsible for the Pinaceae pollen release. With this in mind, the main aim of this study was to indicate the potential origin of the Pinaceae pollen and specifically of *Abies* and *Picea* pollen, recorded in 2013 and 2014 in central Europe. This has been accomplished by an analysis of the meteorological conditions, synoptic situations and back-trajectory analysis computed with the aid of a Lagrangian model. The authors focused on three questions: (1) why *Abies* pollen concentration was observed in such a high quantity in 2013; (2) why Pinaceae pollen was observed before the main pollen season started, and (3) which regions were the possible sources of Pinaceae pollen?

## Materials and methods

### Aeropalynological analyses

The study was performed in Kraków (220 m above sea level, 50°04′N, 19°58′E) located in the Małopolska (Lesser Poland) province of the southern Poland. The city is surrounded by farmlands, especially in the north part, and by forests, especially in the east and west side. Within the territory of Kraków city, parks, squares, boulevards, botanical garden are found. Forest cover constitutes 4.38 % of the total city area (1431 ha), while the Wolski Forest represents the large wooded area on the outskirts of Kraków. The species composition of tree stands in the area is dominated by deciduous species: *Fagus* (20.5 %), *Quercus* (19.0 %), *Betula* (14 %) and *Alnus* (10.1 %) (Chełstowska et al. 2015). Conifers occur less frequently in the city than outside of the agglomeration, especially in its west part, in the Wolski Forest (Zając and Zając 2006).

**Fig. 2:**
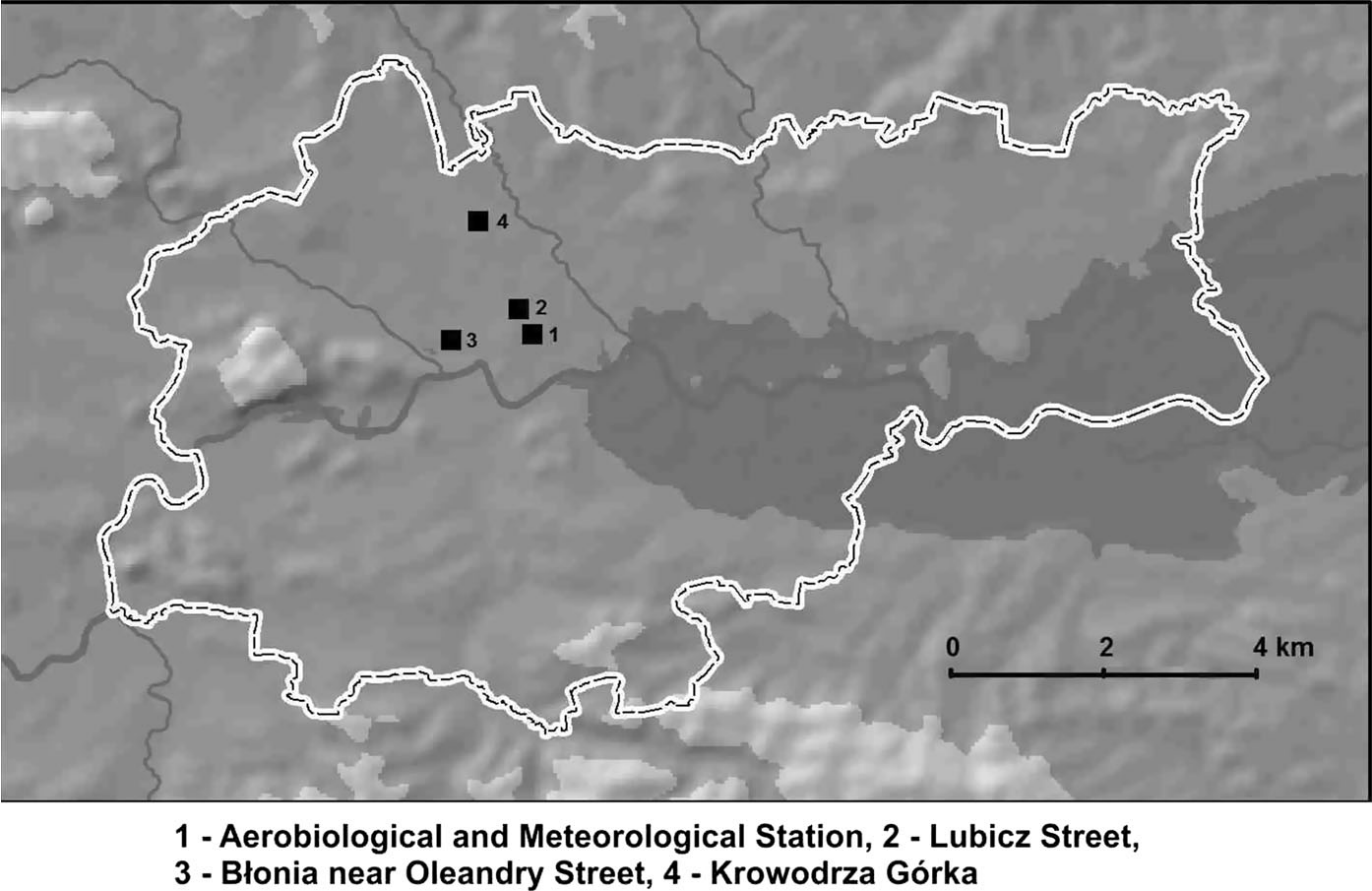
Map of Kraków with the study site locations

**Fig. 3:**
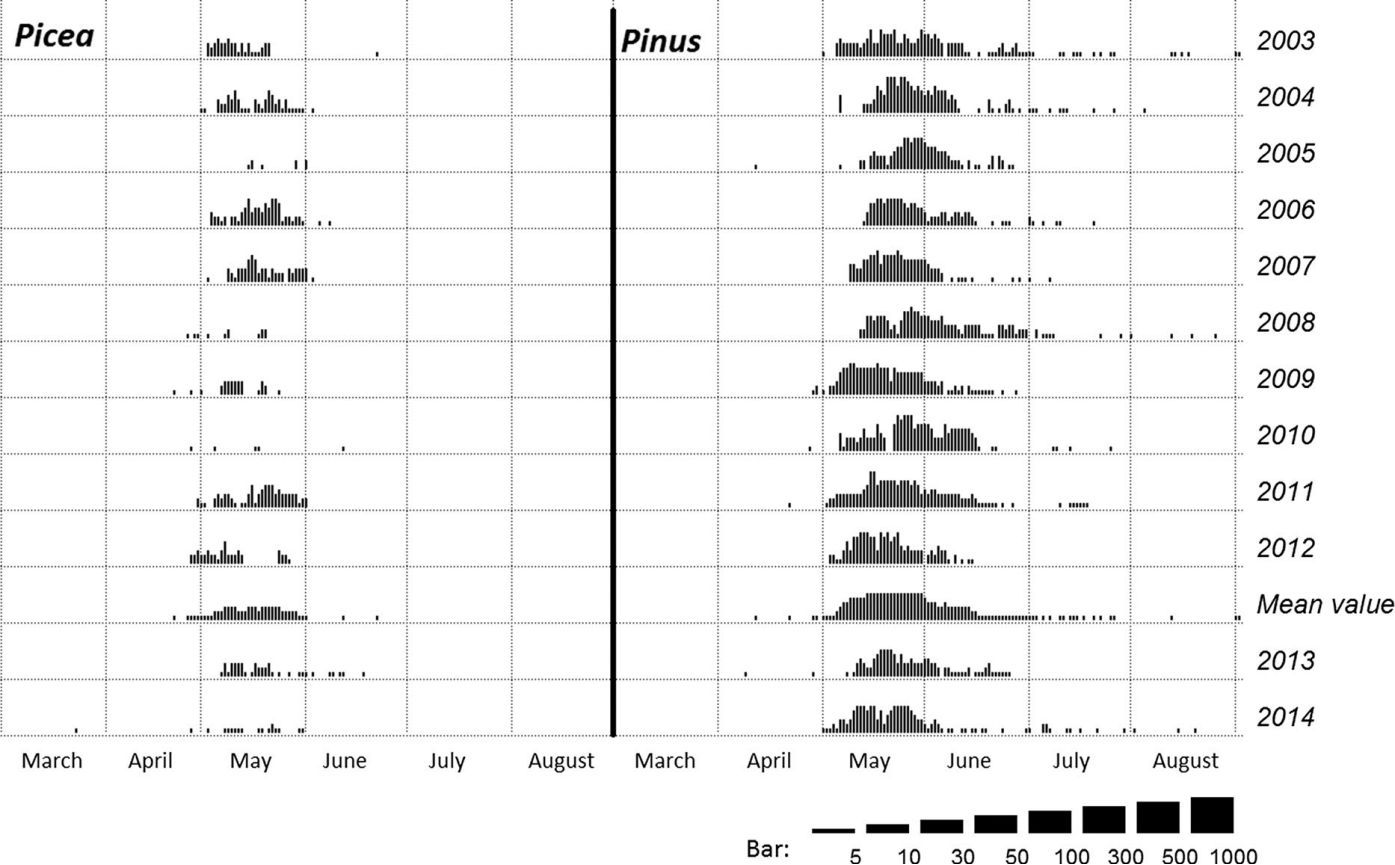
*Picea* and *Pinus* pollen calendars for Kraków, for 2003–2012 and for 2013, 2014

Airborne pollen data were collected using the volumetric method by VPPS 2000 Sampler, Lanzoni Ltd. located on the roof of the Collegium Sniadecki building (site 1) in the city centre, 20 m above the ground level (Fig. 2). Pollen grains were sucked into a rotating drum covered with the transparent tape (*Melinex tape*) coated with an adhesive fluid. Microscope slides were prepared according to the instruction given by Stach and Kasprzyk (2005). The tape with an adhesive fluid was changed once a week and then divided into seven segments corresponding to 24-h periods. The samples were examined using a light microscope at 400× magnification. Pollen grains were counted along 4 longitudinal transects according to the International Association for Aerobiology (IAA) requirements (Galán et al. 2014). Airborne pollen concentrations were expressed as a number of pollen grains per cubic metre (PG m^−3^). Three taxa belonging to the Pinaceae family were taken into account in the current study: *Abies*, *Picea* and *Pinus*. For the purpose of the current study, the data collected in 2013–2014 were used, whereas the data obtained in 2003–2012 were treated as the background data to compare pollen seasons of *Pinus* and *Picea* observed in 2013 and 2014 (Fig. 3). The pollen calendar does not include the *Abies* pollen season because *Abies* pollen was observed only on two occasions, in 2003, May 23 (1 PG m^−3^), and 2007, May 9 (1 PG m^−3^), in the air of Kraków. During the entire study period (2003–2014), *Abies* pollen was only detected in large quantities in 2013.

The first selected period, which was taken into account, was the time of pollen occurrence in the volumetric air samples, with no evident signs of pollen release from local trees (5–12 May 2013). Moreover, high *Abies* pollen concentrations were noted on those days (maximum 35 PG m^−3^). The Pinaceae pollen concentration, shown as a percentage of the daily pollen concentrations of all taxa, was then compared to the surface profiles established on the 13–14 of May 2013 (see Sect. 2.2). In 2014, no occurrence of Pinaceae pollen on the ground surface was noticed, before their detection in the volumetric samples, so the analyses of the airborne pollen concentration and surface profile were compared in one day only (14 May 2014).

Pollen seasons were calculated using the 95 % method, within the start of the season defined as the date when 2.5 % of the seasonal cumulative pollen count was trapped and the end of the season when 97.5 % of the seasonal cumulative pollen count was reached (Jato et al. 2006). The descriptive statistics, like arithmetic mean, minimum, maximum, standard deviation, a coefficient of variation, confidential interval (CI), were applied to define the season characteristics (season start, season end, duration, Seasonal Pollen Index (SPI) value, maximum concentration and the day, when it was achieved). The SPI was calculated as a seasonal total concentration in the determined season.

### Samples from the ground surface

Materials for pollen analysis were collected on May 13 (during the rainfall) and May 14 (after the rainfall) in 2013. During the first day, yellow residue accumulated around puddles in two localities in Kraków: Lubicz Street (site 2) and Błonia near Oleandry Street (site 3) were collected (Fig. 2). The puddles, with water from precipitation, were formed on a smooth and relatively clean surface in shallow depressions on asphalt pavements. These two localities are situated about 2.5 km from each other. After rain, one sample from the pollen scum settling on the bottom and margins of drying puddles was collected also in Kraków at Krowodrza Górka, located about 3 km to the north of both the localities (site 4) (Fig. 2). In 2014, on May 14 (during the rainfall) yellow residue accumulated around puddles in only one locality in Kraków (Lubicz Street) was collected.

The samples were processed in the Laboratory of the W. Szafer Institute of Botany, Polish Academy of Sciences, Kraków, according to the modified Erdtman acetolysis method (Moore et al. 1991). The pollen samples were dehydrated with glacial acetic acid and then heated for about 4 min in a mixture of a sulphuric acid and acetic anhydride. Hydrofluoric acid was not used. The microscope slides were arranged using glycerine or glycerine jelly as a mounting medium. Two microscope slides from each sample were studied using a light microscope at 400× and 600× magnifications. All captured pollen grains were identified and recorded, resulting in more than 2000 pollen grains in total per sample.

### Meteorological data and back-trajectory

The meteorological analysis was based on selected meteorological measurements and observations recorded at the Research Station of the Department of Climatology, Institute of Geography and Spatial Management, Jagiellonian University in Kraków (50°04′N, 19°58′ E, 206 m ASL), which is located in the immediate vicinity of the aerobiological monitoring site. The following meteorological parameters were used: temperature (°C), air pressure (hPa), precipitation (mm), sunshine (h) and cloudiness (%).

In addition, synoptic maps and charts of the geopotential heights of 850 and 500 hPa pressure surface were used, showing the spatial distribution of air pressure, atmospheric fronts and direction of air masses advection. The maps mentioned above were obtained from the Deutscher Wetterdienst Offenbach (DWD; www.1wetter3.de) database. The synoptic maps have a time resolution of 6 h, whereas upper level charts show the structure of the atmospheric baric field at 0:00 and 12:00 UTC.

Moreover, the numeric model Hybrid Single-Particle Lagrangian Integrated Trajectory (HYSPLIT), developed jointly by the NOAA and Australia’s Bureau of Meteorology, was used to compute the paths (so-called back-trajectories) of moving air particles (*NOAA Air Resources Laboratory;* http://www.arl.noaa.gov). The data (with a spatial resolution 1 × 1 degree) used to calculate the back-trajectories come from the operational systems GDAS (Global Data Assimilation System; http://www.arl.noaa.gov), which are often used to study the movement of the air masses. More details regarding the HYSPLIT *model can be found,* among others, in Fernández-Rodríguez et al. (2014; 2015), Hernández-Ceballos et al. (2014b), Jochner et al. (2015).

In each case, trajectories were calculated up to 4 days backwards (96 h), on three levels: 20, 500 and 1000 m above ground level. The lowest level corresponds to the height of the volumetric sampler location, being the closest level to the ground surface. Hernández-Ceballos et al. (2014b) stated that when backward trajectories at low altitudes above ground level (e.g. 20 m) are calculated, some differences in the air stream directions can occur if data of different spatial resolution are used. More specific routes are obtained in the case of data characterized by high spatial resolution. They take into account the changes resulting from the impact of convection, friction forces, geomorphology, etc. The trajectories calculated by using data of slightly lower resolution indicate the approximate direction of the air inflow to a given area.

The transitional level, 500 m ASL, where the air flow is still strongly modified by varied topography of the area is under question. The highest level (1000 m) reflects the free atmosphere, in which the effect of the earth surface friction is disappearing. These heights are usually included in the aerobiological studies (Fernández-Rodríguez et al. 2014, 2015; Hernandez-Ceballos et al. 2014a). The height of 1000 m reflects the circulation of air masses in a free atmosphere and covers areas in the meso- and macroscale. Pollen grains detected at that height most likely do not originate from local sources, but from the long-distance transport. This height has been also taken into consideration, because of the barrier of the Carpathian and the Sudeten Mountains. Pollen grains transported from southern Europe most likely reach this height to move above the mentioned mountain ranges. At a height of 500 m, pollen grains can come from both long-distance transport and local sources. They may in fact be gained at this height as a result of movements of convection and turbulence.

The choice of the 96-h period, for which the trajectories were calculated, resulted from the:

- need to check trajectories of considerable length;
- time needed to perform the transformation of the air mass (minimum of 3 days);
- changes in atmospheric circulation taking place over the relevant area;
- possibilities of individual pollen taxa transport over long distances;
- successive fall of pollen during a long-range transport.

## Results

### Pinaceae pollen seasons in Kraków

Pine and spruce pollen seasons in Kraków in 2003–2014 differ slightly in a course of the season (Fig. 3). In the case of *Picea*, the pollen seasons are less intense and could be described as “time of pollen occurrence” rather than the “pollen seasons”. *Pinus* pollen seasons are more intense; in most years, the pollen concentration increases rapidly, while the more gentle pollen decreases with the single grains occurring up to 2 months after the main pollen seasons were observed. The pollen calendar does not include the *Abies* pollen season, which has been already explained in Materials and Methods section.

The pollen seasons were stable for a 10-year period (2003–2012), and the coefficient of variation ranged from 3.8–4.4 % to 4.7–5.3 % (season start and season end, respectively) (Table 2). Pinaceae pollen occurs in May, up to 4 weeks achieving different SPI values in consecutive years. The maximum *Pinus* pollen concentration was achieved, on average, 1 week after the season start, and correlated significantly with the season start date (*r*_*s*_ = 0.817) and season end date (*r*_*s*_ = 0.744). The maximum concentration also correlated with the SPI value (*r*_*s*_ = 0.867). In the case of *Picea*, the only statistically significant correlation was found between the SPI value and maximum concentration (*r*_*s*_ = 0.948). *Pinus* and *Picea* pollen season characteristics in 2013 were similar to the previous years (2003–2012), but calculating season characteristics in 2013 we did not exclude pre-seasonal pollen occurrence (Table 2). In the case of *Abies*, the evident high pollen concentrations were noted in 2013 (pollen sum in 1–14 May equals 128 PG m^−3^). In 2014, the airborne *Pinus* pollen concentration was higher than atmospheric concentrations of *Picea* pollen, and *Abies* pollen was not observed (Table 2). The Pinaceae pollen seasons started in the first week of May; the curve of *Pinus* pollen dynamics indicated the periods of higher pollen concentrations, between which the pollen decreases in the middle of May, caused by a presence of rainfall (Fig. 5).

**Table 2:** Descriptive statistics of some *Picea* and *Pinus* season characteristics in Kraków, in 2003–2012, and the pollen season characteristics in the studied years (2013–2014)

Descriptive statistics	Season characteristics																	
	*Picea*									*Pinus*								
	Season start^1/2^		Season end^1/2^		Season duration^3^	SPI^4^	Max. conc^4^	Day of max.^1/2^		Season start^1/2^		Season end^1/2^		Season duration^3^	SPI^4^	Max. conc.^4^	Day of max.^1/2^	
Min	116	26/04	138	18/05	18	7	2	127	6/05	121	1/05	144	24/05	21	1259	157	122	2/05
Median	122	2/05	146	25/05	24	243	48	134	14/05	130	10/05	156	5/06	29	3340	459	137	18/05
Max	133	13/05	161	10/06	46	768	146	140	19/05	136	15/05	175	23/06	43	5062	956	147	27/05
$\bar{x}$	122	2/05	146	25/05	25	287	57	134	14/05	130	10/05	157	6/06	28	3323	498	137	17/05
SD	5.4	–	6.9	–	8.1	251.5	52.5	4.3	–	4.9	–	8.3	–	6.4	1358.5	242.8	8.2	–
*V* %	4.4	–	4.7	–	32.1	87.5	92.3	3.2	–	3.8	–	5.3	–	22.8	40.9	48.8	5.9	–
−95 %;	118.1	–	141.4	–	19.5	107.5	19.4	130.9	–	125.9	–	150.7	–	23.5	2351.5	323.8	130.8	–
95 %	125.8		151.2		31.1	467.3	94.5	137.1		133.0		162.5		32.7	4295.1	671.2	142.5	
2013	126	6/05	157	6/06	32	164	24	128	8/05	130	10/05	164	13/06	35	1155	135	138	18/05
2014	116	26/04	149	29/05	34	31	9	140	20/05	127	7/05	152	1/06	26	2445	273	131	11/05

^1^Following day from the 1 January; ^2^date; ^3^number of days; ^4^ PG m^−3^—to long pause pollen grains in 1 m^3^ of air, *SPI* Seasonal Pollen Index

### Pinaceae pollen falls on selected days in 2013 against a background of meteorological conditions and back-trajectories analysis

**Fig. 4:**
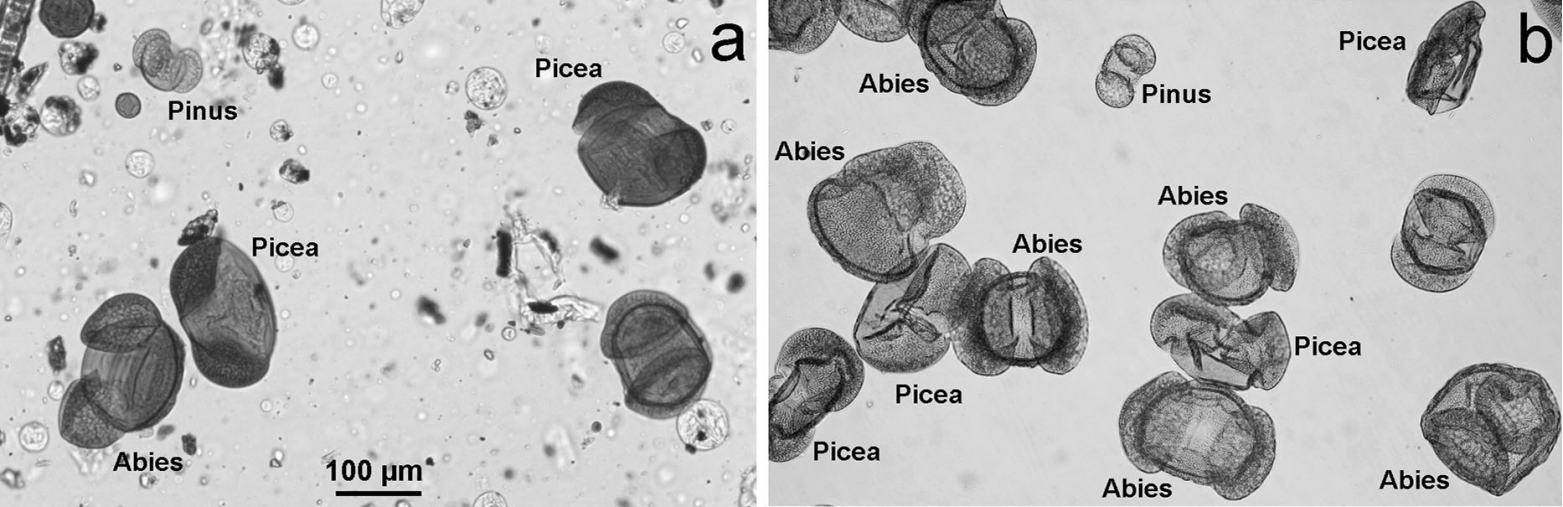
Pictures of pollen occurrence in the volumetric samples (**a**) and the samples collected from the surface (**b**)

Yellow residue accumulated around puddles was composed almost exclusively of pollen grains (Fig. 4). In the two samples collected during the rainfall on the 13 May 2013, three taxa of gymnosperms dominated: *Abies* (65.29 % in the sample 3 and 66.37 % in the sample 2), *Picea* (25.72 % and 23.95 %, respectively) and *Pinus sylvestris* type (8.55 and 8.75 %, respectively). Pollen of the other taxa occurred subordinately or sporadically (Table 3). In the third sample, collected the day after the rainfall (14 May 2013) the same three taxa of gymnosperms also dominated the combined pollen of *Abies* (43.15 %), *Picea* (28.12 %) and *Pinus sylvestris* type (7.44 %) made up 78.71 % of the pollen spectrum (Table 3). In comparison, the percentage of *Abies* and *Picea* pollen in the volumetric samples in 13–14 May 2013 was definitely lower, while the percentage of *Pinus* pollen was higher, but other taxa dominated in both days (Table 3).

**Table 3:** The percentage of the pollen content of determined taxa in the samples collected on the surface and in volumetric samples, in the studied days in 2013 and 2014

Type of method	Samples from the surface				Volumetric method		
Data	13/05/2013		14/05/2013	14/05/2014	13/05/2013	14/05/2013	14/05/2014
Taxon/place of collection	Site 3	Site 2	Site 4	Site 2	Site 1		
Studied taxa							
*Abies*	65.29	66.37	43.15	0.05	–	4.17	–
*Picea*	25.72	23.95	28.12	1.18	–	1.39	–
*Pinus sylvestris* t.	8.55	8.75	7.44	98.77	15.00	44.45	86.67
Other taxa	0.44	0.93	21.29	–	85.00	49.99	13.33

**Fig. 5:**
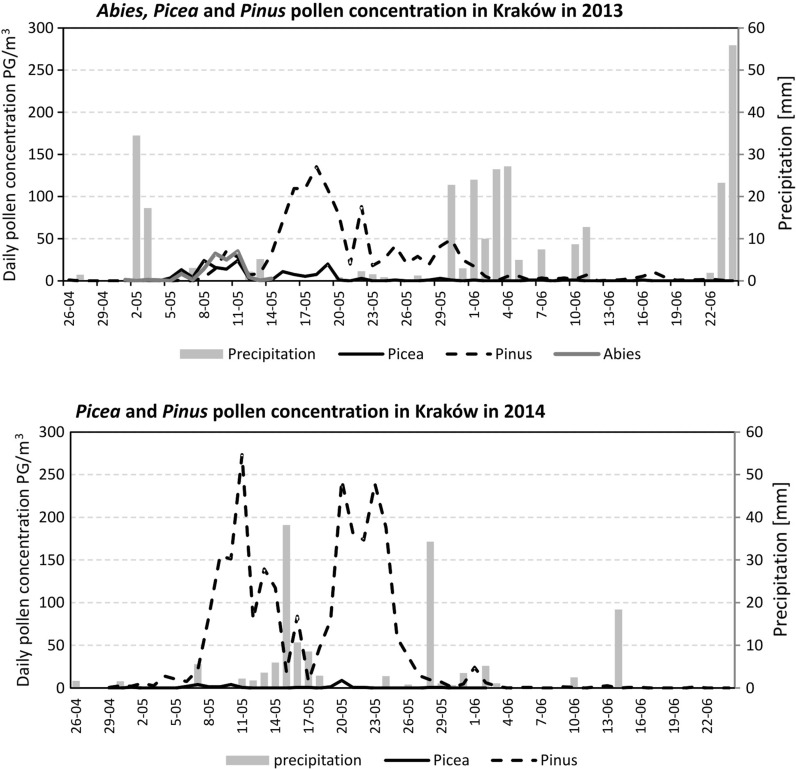
Pinaceae pollen occurrence in comparison with daily precipitation totals or "total precipitation" in Kraków in 2013 (including *Abies*, *Picea* and *Pinus* pollen) and 2014 (*Picea* and *Pinus* pollen)

In 2013, it is seen that the first period of higher *Picea* and *Pinus* pollen concentrations was observed from the 5 to 12 of May, earlier than the main pollen season started (Fig. 5). *Abies* pollen was also observed in these days. Rainfall detected on the 12 and 13 of May, caused the pollen deposition on the ground surface. In order to explain an unusually early pollen appearance of the studied taxa, weather conditions before and during the period of the pollen occurrence were analysed.

**Fig. 6:**
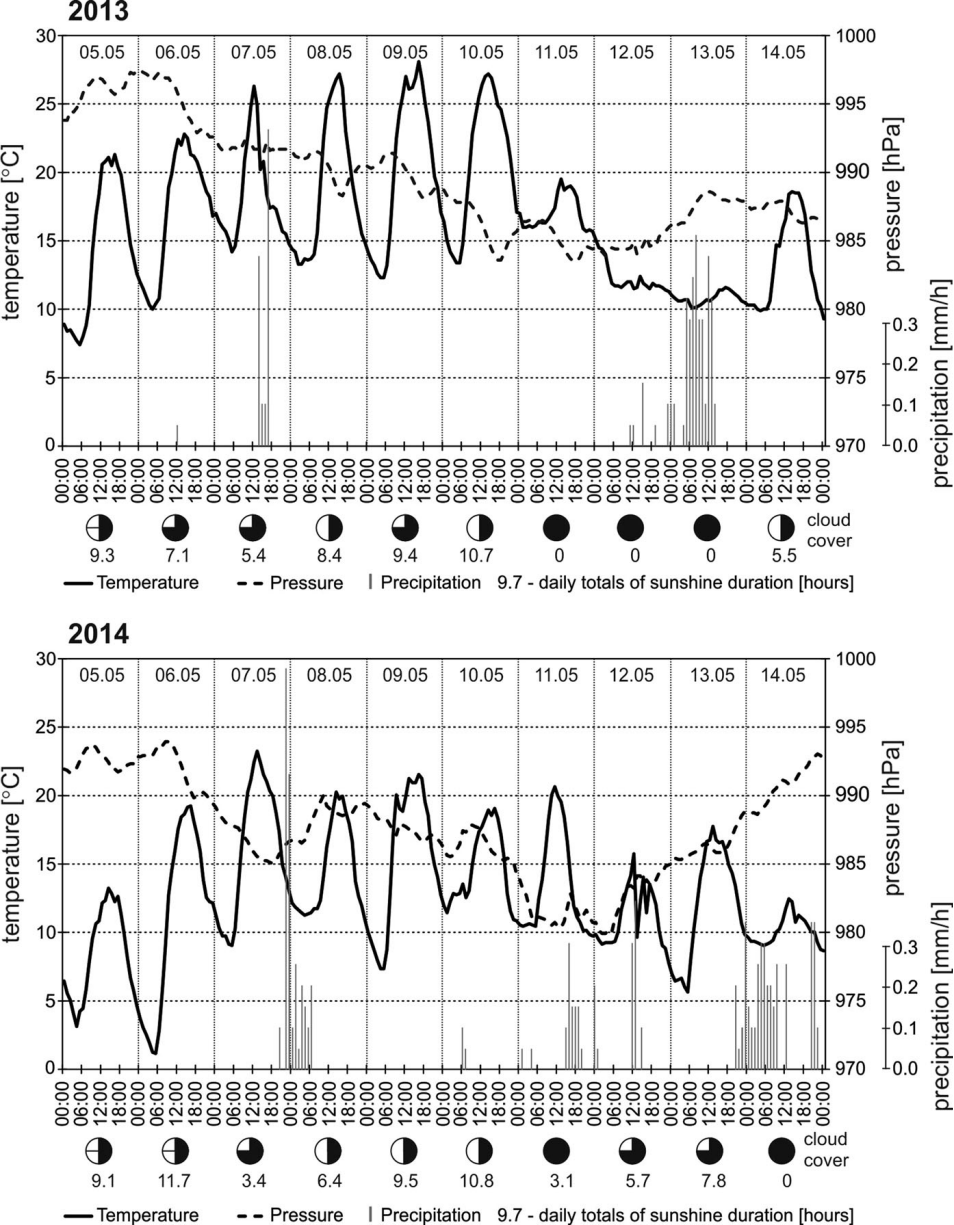
Daily course of air temperature, air pressure, precipitation and sunshine duration in Kraków on 5–14 May 2013 and 2014

Since mid-April 2013, almost the entire central European region located below 55°–60°N was influenced by anticyclonic systems, often forming a zone of high pressure extending from the North Atlantic to Russia. In Kraków, during this period the maximum air temperature was mostly above 20 °C. In early May, high-pressure area moved from the Atlantic to the east. The periphery of that area, in Kraków, was occupied by the quasi-stationary front, which contributed to the deterioration of the weather. It was cloudy, quite cool (Tmax 13–18 °C) and with intense precipitation (34 mm) on the 2 and 3 of May (Fig. 5). In the following days, a zone of high air pressure extended over the area examined. It brought about an increase in temperature (Tmax to 28 °C) and sunshine up to over 5 h (Fig. 6).

On 4–7 May 2013, the high-pressure system covering almost all Europe was slowly moving north-east covering Latvia, Lithuania, Estonia, Belarus and the western part of Russia. During the next day, anticyclone mentioned above connected with a high-pressure system from Scandinavia and as a belt of high-pressure covered East Europe and the European part of Russia up to the 10 May. At the same time, from the 6 May to the 7 May a warm front connected with a cyclone developed over the Italian Peninsula and, accompanied by precipitation, approached Poland. During the next few days, subsequent fronts moved over Poland. However, a blocking high-pressure centre located to the east of Poland stopped Atlantic air masses over the western part of the country and caused the influx of very warm air masses from the southern part of Europe.

From 9 to 10 May, a high-pressure system broke up into a weak high over Scandinavia and another high that moved beyond the Black Sea. The air from the south still moved into Poland. In the following days, the low-pressure system reached Poland bringing precipitation in Kraków on the 12 and 13 May 2013 and the subsequent change in atmospheric circulation.

**Fig. 7:**
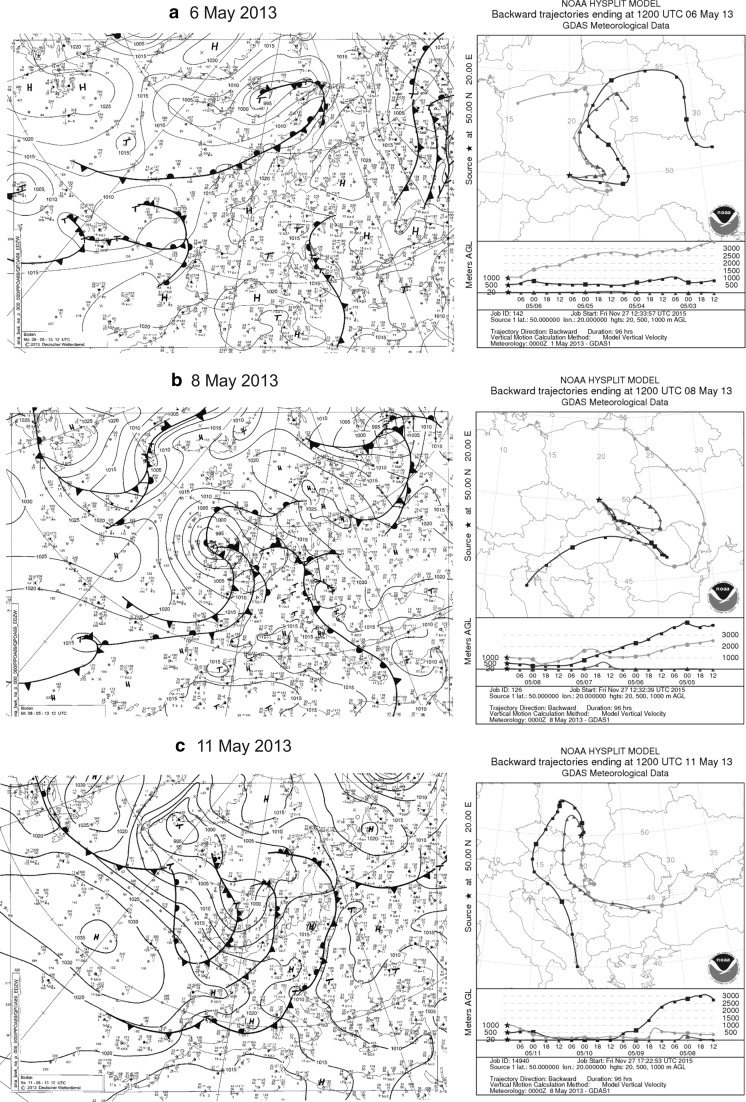
Synoptic maps and 4-day backward trajectories of air particles in the periods: **a** 3–6, **b** 5–8 and **c** 8–11 May 2013 on three levels: 20, 500 i 1000 m a.g.l

The analysis of the synoptic situation shows that in the first days of May (4–7 May; see above) in areas under the influence of the described high, sunny weather prevailed. In the regions located to the south and south-east of Poland, temperatures were high (up to 30 °C) and conditions were favourable for *Abies* and *Picea* pollination. However, air masses lying over Kraków were much colder during 4–6 May, preventing the start of the pollen season of the studied taxa. In spite of weather conditions not being favourable, pollen of *Abies* and *Picea* appeared in Kraków. The detailed analysis of synoptic maps and the back-trajectories suggested that pollen grains could have been transported from Ukraine and Slovakia, a distance of 500–750 km. This was due to the direction of air flow. At the beginning, air masses approached from the North and then Ukraine and Slovakia before finally reaching Kraków (Fig. 7a).

After a small amount of rain on the 6 and 7 May, when the most of the pollen grains were washed out from the air, the airborne Pinaceae pollen concentration increased once again. Since *Abies* and *Picea* were still not flowering, the pollen grains collected must have originated from the long-distance transport. The analysis of air masses and back-trajectories indicates that they probably flew from Ukraine, Romania, Hungary and Slovakia, and even from the Balkan Peninsula (~1000 km; Fig. 7b–c). Atmospheric concentrations of pollen decreased from the 12–14 of May, which was associated with a circulation change and deposition from rainfall. The only increases in airborne pollen concentrations that can be attributed to trees growing in the vicinity of Kraków, especially of *Pinus*, were recorded during the in the second half of May 2013.

### Pinaceae pollen falls on selected days in 2014 against a background of meteorological conditions and back-trajectories analysis

The composition of the pollen spectrum in the sample collected 14 May 2014 was different in comparison with 2013 (Table 3). The assemblage was strongly dominated by *Pinus sylvestris* type (98.77 %), whereas only sparse pollen grains of *Picea* (1.18 %) and single pollen grains of *Abies* (0.05 %) were recorded. In the case of the volumetric sample, *Pinus* pollen also prevailed in the pollen spectrum on the 14 May 2014.

The described cycle of pollinating species was favoured by the weather prevailing over the study area. In spring 2014, the contribution of the high-pressure systems affecting the weather in the studied region was slightly lower than in 2013. In the second half of April 2014, the weather in Central and Eastern Europe was affected by the frequent passing of low-pressure systems. These baric systems, that cause airflow from the northern sector, were separated by the high-pressure system. They were accompanied by a slightly cloudy weather and temperatures up to 20 °C.

**Fig. 8:**
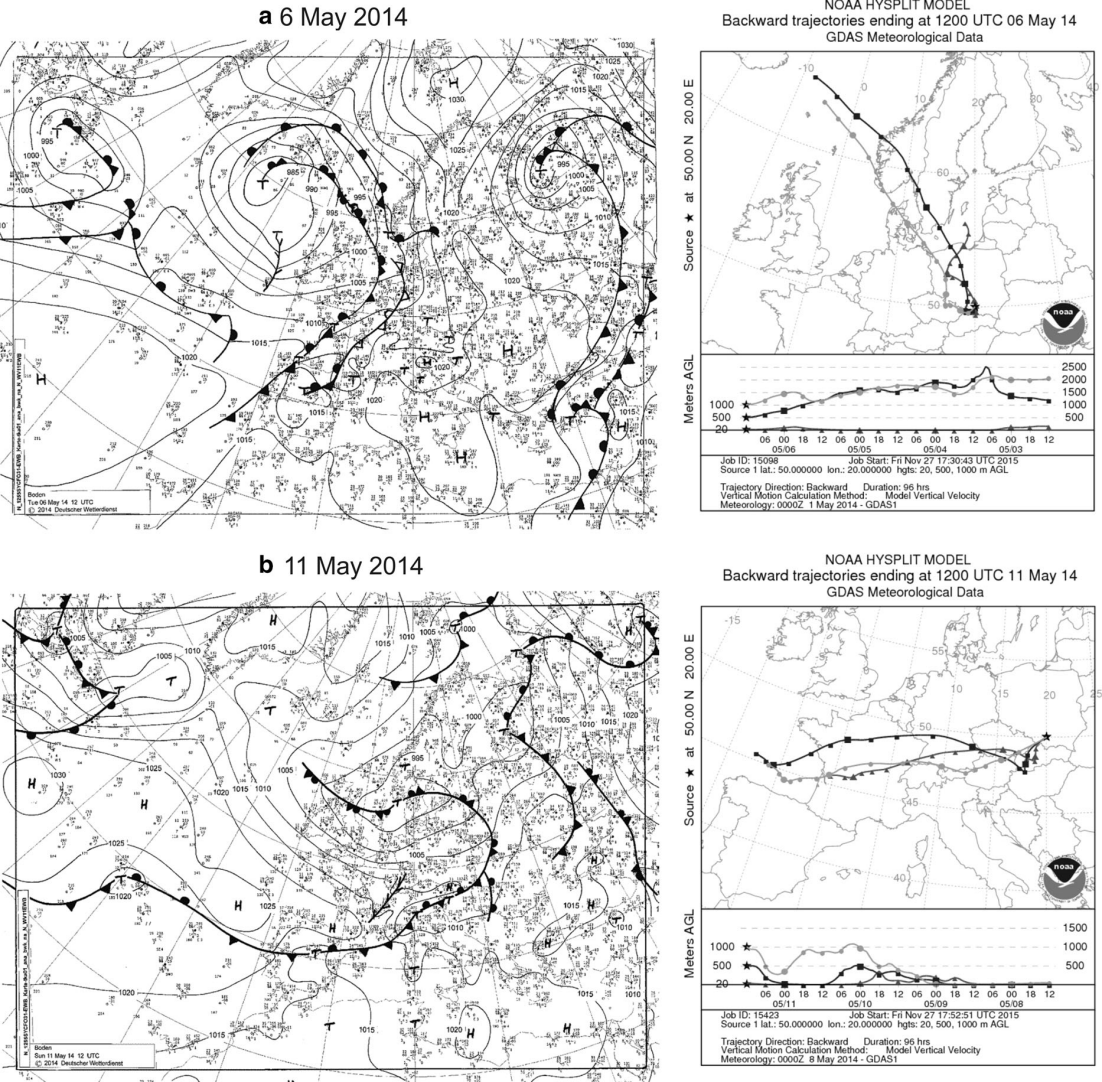
Synoptic maps and 4-day backward trajectories of air particles in the periods: **a** 3–6 and **b** 8–11 May 2014 on three levels: 20, 500 i 1000 m a.g.l

At the beginning of May 2014, similar weather (Fig. 6) influenced the start of the Pinaceae pollen season in central Europe, including Poland (Fig. 5). Pollen grains originated from the local sources, as confirmed by the back-trajectory analysis, indicated the air masses arrived from the North where tree vegetation have not yet started (Fig. 8a).

Since the 7 May 2014, the weather in central Europe was influenced by low-pressure systems frequently moving from west to east accompanied by precipitation. These lows were separated by short periods of sunny and relatively warm weather (Fig. 6). Precipitation limited the pollen concentration, while warm and sunny weather resulted in higher levels of atmospheric pollen (Fig. 5). Strong westerly flow, confirmed by the back-trajectories, indicates the air masses flowed to Kraków from the regions of low airborne Pinaceae concentrations (Fig. 8b). Nevertheless, it is possible that some of the pollen grains arrived from the regions located towards the south-west of Poland (Fig. 8b).

## Discussion

Dust, different aerosols and biological components of the air have been described several times as air mass tracers both in studying atmospheric transport processes and in palaeo-climatological analyses (Manecki et al. 1978; Kellogg and Griffin 2006; Makra and Pálfi 2007 and publications cited herein). Studies of the airborne pollen occurrence and deposition are among investigations that aim at an interpretation of fossil pollen diagrams and are of great importance since they provide the tools to reconstruct past environments (Giesecke et al. 2010). Conifers belong to the group of trees searched intensely by palaeo-ecologists in terms of their pollen presence/absence threshold values. These values reflect the long-distance transport of pollen to areas beyond the distribution range of tree species and are used in tracing the spread of *Abies*, *Picea* and *Pinus* after the last glaciation (Bourgeois et al. 2000; Hicks 2001).

The occurrence of conifer pollen on the ground is related to some atypical meteorological situations and anthropogenic activity, like the long-distance transport of soil, pollen and stable organic compounds in the northern Fennoscandia in March 1991 as yellow-snow episode reported by Franzen et al. (1994). They concluded that from a palaeo-ecological point of view, long-distance transport such as this should be considered as a potentially important source of error whenever interpreting Holocene pollen diagrams. According to Środoń (1960), the presence of pollen derived from long-distance transport in Hornsund (West Spitsbergen) is probably caused by the movements in the upper strata of the atmosphere. The inclusion of trees pollen from long-distance transport in Hornsund is fairly constant, but in the peat samples it is higher than that established in the contemporary surface samples collected at more or less the same altitudes. Studies of pollen transport, including Pinaceae, may prove useful when interpreting pollen diagrams illustrating the vegetation, which overgrew the periglacial zone at the time of the Pleistocene glaciation (Środoń 1960). Bourgeois et al. (2001) analysed the spatial patterns of pollen deposition in arctic snow, which indicated that pollen percentages and concentrations were related to the density of the regional vegetation and to the distance to the source of more productive regions. Pine is particularly valuable in this sense because it has longer trajectories than other tree pollen.

Understanding the relationship between pollen occurrence and vegetation can be provided by using the modern pollen rain in different regions. The results obtained by Autio and Hicks (2004), who analysed annual variation in meteorological parameters and modern pollen deposition in Finland, suggested that annual quantities of fossil *Pinus* pollen, if calculated from peat and/or lake sediments, are a potential climate proxy. The authors of a recent paper examined the long-distance transport *Ambrosia artemisiifolia* pollen, which indicated that the most likely sources of pollen that arrived in south-eastern Poland are Ukraine and Slovakia (Kasprzyk et al. 2011).

The other aspect of the Pinaceae pollen transport is the difference in the flying abilities of pollen grains between the three studied taxa. *Abies* pollen grain is much bigger and has a deposition rate than *Pinus* pollen grains. According to Szczepanek (2003) after Dyakowska (1937), the speed of pollen descent in stable air is as follows: *Abies pectinata* (38.71 cm s^−1^), *Picea abies* (8.7 cm s^−1^) and *Pinus sylvestris* (2.5–3.96 cm s^−1^). The study performed by Robledo-Arnuncio (2011) assessed wind pollination over scales closer to the maximum observed physical pollen transport distances. The results revealed significant effective pollen flow (up to 4.4 %) from a large population of *Pinus sylvestris* located 100 km away, suggesting that the well-known mesoscale airborne transport of viable pine pollen can result in successful pollination over larger scales than previously reported for wind-pollinated tree species.

On the other hand, the problem of the long-distance transport of Pinaceae pollen is also investigated by the aerobiologists and palynologists dealing with modern “pollen rain”. Among others, they are mainly interested in the allergenic taxa, while Pinaceae pollen is treated as low allergenic. For this reason, Pinaceae pollen is frequently determined as a family, in the majority of aerobiological papers (Clot 2003; Peternel et al. 2003). However, from the author’s point of view, the simultaneous analyses of the particular genera (species) are of a great importance. The occurrence of pollen of a given genus of Pinaceae in the air is determined by the local vegetation, different speed of pollen deposition and meteorological conditions. Autio and Hicks (2004) indicated that, within the forest and at the physiognomic forest line, *Pinus* pollen deposition is primarily from plants growing within the forested area on the fell and that the contribution of wind-blown pollen from further south of Finland is minimal. In spite of that, *Pinus* sp. pollen is a very common component of long-distance transported pollen, its occurrence in the air is first of all related to the local sources, while the abundant occurrence of *Abies* and the presence of *Picea* pollen in the “yellow rain” are a rare phenomenon in Poland, although it depends on a region.

The authors agreed that pollen production of *Abies* and *Picea* differs strongly from year to year (Huusko and Hicks 2009; Pidek et al. 2015). Variations in amounts of *Picea* pollen recorded in Kraków 2003–2012 is comparable with pollen monitoring data in the Roztocze region (SE Poland), where all three genera *Pinus*, *Picea,* and *Abies* are important components of the forests. There is an interesting coincidence of the years of higher *Picea* pollen values in Kraków (in 2003, 2004, 2006 and 2007 years) with those recorded in the Roztocze region (Pidek et al. 2015). A 13-year time series of monitoring the annual pollen deposition of fir revealed great inter-annual variability, among which are 2 years (2004 and 2010) of very high pollen deposition in Roztocze (SE Poland), (Pidek et al. 2013). That is why it is worth noting the unusual situation in Kraków in May 2013, because of the lack of *Abies* pollen records in other seasons. Due to the fact that the regular pollen observations in Kraków (throughout a period of more than 20 years) indicated certain regularities of Pinaceae pollen seasons (Myszkowska et al. 2014), the most probable association of the pre-season pollen occurrence with the pollen transport into Kraków was noted evidently.

To the weather conditions, influencing the dispersal of airborne pollen grains belongs: wind direction, wind velocity, temperature, relative humidity, precipitation, pressure and solar radiation, and on the micro- and macrotopography within the area, where the transport takes place, like in case of *Betula* pollen reported by Hjelmroos (1991). Smith et al. (2008) stressed that *Ambrosia* pollen transport to Poland was related to the deep of PBL (*planetary boundary layer*), the passage of air masses over the Carpathian and Sudeten Mountains and it was associated with hot, dry weather and winds from the south. Huusko and Hicks (2009) found on the basis of 23-year observations of spruce and pine pollen deposition (pollen accumulation rates: PARs) the high correlation between pollen quantity and summer temperature around July in the year before the pollen emission. The similar observations were published by Green et al. (2003), who found a statistically significant correlation between daily average airborne *Pinus* pollen concentrations and minimum, maximum temperature and rainfall, while Hjelmroos (1991) indicated the influence of air turbulences and washout on *Betula* pollen fall in Scandinavia. For *Pinus sylvestris*, the quantity of pollen deposited is affected by mean July temperatures, July effective temperature sum and total effective temperature sum, for the year previous to the pollen emission (Autio and Hicks 2004).

The other question is where the pollen grains come from? Looking on the maps of the Pinaceae plant distribution and analysing the synoptic situation in Europe at the beginning of May 2013, the following regions were indicated as the most evident sources of pollen: south-eastern Hungary, central Romania and Balkan Peninsula in case of *Abies* pollen; Slovakia, central Romania, Ukraine in case of *Picea* pollen; and Slovakia, central Romania, Ukraine and the Balkan Peninsula in case of *Pinus* pollen. The sunny, warm weather with little rainfall dominated at that time over these regions, causing the earlier start of Pinaceae pollination in areas located in the south and east of Poland.

This is confirmed by the fact that at the end of April and in the first week of May 2013, Pinaceae pollen was observed in Serbia (personal communication). The information on *Pinus* pollen season in Zagreb (Croatia) in 2002, published by Peternel et al. (2003), showed that pollen was detected from the beginning of May to the middle of June. In contrast, the yellow rain of Pinaceae pollen in May 2014 originated from the local trees. The analysis of the back-trajectory confirmed westerly and north advection, from the regions of low Pinaceae occurrence and later pollination.

## Conclusions

In the current study, Pinaceae pollen (particularly *Abies* and *Picea* pollen) found in the aerobiological samples and on the surface of puddles (“yellow rain”) at a time when no local trees were pollinated reached Kraków from the distant sources.

*Abies* pollen is only occasionally recorded in Kraków. It has been confirmed that the occurrence of this pollen type in Kraków in 2013 was due to long-distance transport. The presented episodes of the long-range transport of pollen confirmed this phenomenon, for example, of Pinaceae pollen, which can result in spectacular pollen fall (“yellow rain”) beyond the actual pollen season. It is also worth noting that back-trajectory analysis is a useful tool for distinguishing the local pollen occurrence and the long-distance transport episodes, as confirmed by the Pinaceae pollen situation in 2014 in Kraków.
